# Graphene quantum dots mediated magnetic chitosan drug delivery nanosystems for targeting synergistic photothermal-chemotherapy of hepatocellular carcinoma

**DOI:** 10.1080/15384047.2022.2054249

**Published:** 2022-03-24

**Authors:** Lili Chen, Wenzhong Hong, Siliang Duan, Yiping Li, Jian Wang, Jianmeng Zhu

**Affiliations:** a Department of Orthopedics, Chun’an First People’s Hospital, Zhejiang Provincial People’s Hospital Chun’an Branch, Hangzhou Medical College Affiliated Chun’an Hospital, Hangzhou, P.R. China; b Clinical laboratory of Chun’an First People’s Hospital, Zhejiang Provincial People’s Hospital Chun’an Branch, Hangzhou Medical College Affiliated Chun’an Hospital, Hangzhou, P.R. China; c The department of Immunology, Medical College, Guangxi University of Science and Technology, Liuzhou, P.R. China

**Keywords:** Hepatocellular carcinoma, drug delivery nanosystems, combined therapy, magnetic chitosan, graphene quantum dot

## Abstract

Conventional clinical monotherapies for advanced hepatocellular carcinoma (HCC) have numerous limitations. Integrated oncology approaches can improve cancer treatment efficacy, and photothermal-chemotherapy drug delivery nanosystems (DDS) based on nanotechnology and biotechnology have piqued the interest of researchers. This study developed an aptamer-modified graphene quantum dots (GQDs)/magnetic chitosan DDS for photothermal-chemotherapy of HCC. The HCC aptamer and the EPR effect of nanoparticles, in particular, enable active and passive targeting of DDS to HCC. GQDs functioned as photosensitizers, effectively moderating photothermal therapy and inhibiting drug release during blood circulation. Magnetic chitosan demonstrated excellent drug encapsulation, acid sensitivity, and tumor imaging capabilities. Proper assembly of the units mentioned above enables precise combined therapy of HCC. This study indicates that DDS can significantly inhibit tumor growth while also extending the survival duration of tumor-bearing mice. The DDS (DOX-Fe_3_O_4_@CGA) shows strong synergistic tumor treatment potential, allowing for the exploration and development of novel HCC therapies.

## 1. Introduction

HCC is one of the most common types of cancer globally.^1^ Advanced HCC is generally difficult to treat.^2^ Due to inefficiency and toxic side effects, single therapies such as chemotherapy and radiotherapy cannot produce sufficient therapeutic benefits.^3^ As a result, it is critical to developing combined treatments to overcome the drawbacks of conventional treatments and improve the overall effect.^4,5^

Because of its high specificity and minimally invasive advantages, photothermal therapy (PTT) has recently been a research hotspot among therapeutic techniques for HCC.^6^ Photothermal therapy employs a light source with good tissue penetration to irradiate the tumor site. The photosensitizer can transform light energy into heat energy, allowing thermal ablation of the tumor site to be performed.^7^ Compared to traditional therapy, studies reveal that photothermal therapy offers a shorter treatment time, an apparent curative outcome, and less biological toxicity.^8^ More importantly, photothermal therapy and traditional chemotherapy have a strong synergistic effect and therapeutic transformation potential.^9^ The heat generated by photosensitizers can boost the uptake rate of drug carriers by tumor cells, shorten the release time of chemotherapy drugs, avoid drug resistance, and improve chemotherapy efficacy.^10^ However, precisely targeting chemotherapeutic drugs and photosensitizers to the tumor site simultaneously and managing drug release to obtain the maximal therapeutic effect while avoiding side effects is the key to effective HCC treatment.^11,12^

In recent years, nano-drug loading technology has demonstrated distinct advantages in tumor treatment. Nanocarriers with 10–200 nm diameter enter tumor tissues without being delivered there by lymphatic system reflux.^11,13^ They were concentrated in tumor tissues because of the high permeability and long retention effect (EPR effect). Hydrophobic drugs that are water-insoluble can be encased in nanoparticles to make them more water-soluble and avoid the negative effects of standard cosolvents.^14,15^

Because of their positive charge on the surface and mucosal viscosity, chitosan (CS) drug-loaded materials easily penetrate the cell membrane, making them one of the best nano-drug carriers.^16^ Chemotherapy drugs, such as doxorubicin (DOX), create a chemical bond (N-C-) with CS via the aldehyde group, which allows the drug to remain active for an extended period. *In vivo*, chitosan does not induce allergic reactions or rejection and can be metabolized into harmless monosaccharides absorbed by the human body.^17,18^ Many active amino groups in the molecules are easily cleaved in the low pH tumor microenvironment. As a result of its bioadhesive, biodegradable, and pH-responsive nature, CS is a good option for DDS.^19,20^

Furthermore, nanoparticles such as quantum dots, magnetic iron oxide particles (γ-Fe_2_O_3_, Fe_3_O_4_), and polymers can be easily added to the surface of CS. According to studies, small-sized CS-coated magnetic Fe_3_O_4_ can absorb more drug molecules and increase the dispersion and stability of nanoparticles.^21^ Due to its superparamagnetism and good biocompatibility, magnetic nano-sized iron oxide offers significant application potential in constructing tumor-targeted MRI imaging probes and DDS.^22,23^

Photosensitizers are essential for achieving effective photothermal therapy. Traditional photosensitizers, such as noble metal nanoparticles and indocyanine green (ICG), have numerous flaws. Metal materials are frequently poisonous, difficult to break down, and harmful to organisms.^24^ Although ICG photosensitizer can deteriorate *in vivo*, it only has a limited thermal effect, and it is not easy to promote drug release. GQDs, being carbon-based quantum dots, offer outstanding optical absorption qualities as well as low cytotoxicity and great biocompatibility.^24,25^ When exposed to 808 nm near infrared (NIR) light, the conjugated π bond of GQDs absorbs photons. It transforms them into heat, promoting rapid heat of the surrounding environment and producing reactive oxygen species (ROS) to accelerate tumor cell death.^26^ Furthermore, the surface functional groups (hydroxyl, epoxy, and carboxyl) of GQDs allow them to interact with a wide range of nanoparticles, nucleic acids, and proteins.^27^ GQD appears to be a promising photosensitizer.

**Scheme 1.**
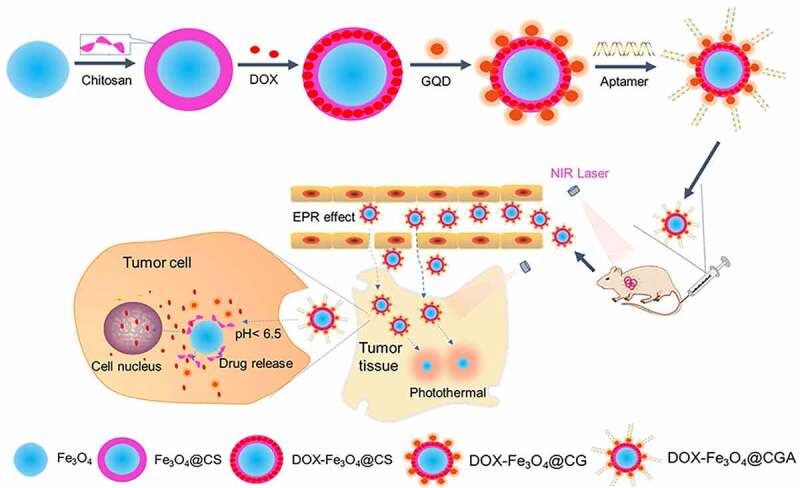
Schematic illustration of the synthesis of DOX-Fe_3_O_4_@CGA and its targeting and synergistic chemo-photothermal therapy of HCC.

Abbreviation: CS, chitosan; CG, Chitosan@GQD; CGA, Chitosan@GQD@Aptamer; DOX, Adriamycin; NIR, Near-infrared light;

GQDs modified magnetic chitosan chemo/photothermal DDS were produced in this study. As illustrated in Scheme 1, magnetic Fe_3_O_4_ nanoparticles were created using the co-precipitation approach. Lin et al. described the method of CS production by coating magnetic iron with CS.^28^ To preserve pharmacological action, DOX was loaded into CS via the aldehyde group, and GQDs were changed on the surface of magnetic CS via an amide link. Finally, the TLS11a aptamer with a strong affinity for H22 mouse liver cancer cells^29^ was employed to functionalize the nanoparticles, resulting in DOX-Fe_3_O_4_@CS@GQD-Apt (DOX- Fe_3_O_4_@CGA). The DDS can particularly target HCC and has pH sensitivity. Experiments *in vivo* and *in vitro* revealed that the DDS effectively treats tumors with synergistic photothermal-chemotherapy, establishing a potential nano platform for HCC therapy.

## 2. Materials and methods

### 2.1 Reagents

Citric acid(Cat.No:251275), FeSO_4_ · 7H_2_O(Cat.No:215422), PEG-2000(Cat.No:8.21037), Chitosan(CS) (Cat.No:448869) 1-ethyl-3-[3-dimethylaminopropyl] carbodiimide hydrochloride (EDC) (Cat.No: 341006), and N-hydroxysuccinimide (NHS) (Cat.No: 8.04518) were purchased from Sinopharm Chemical Reagent Co., Ltd; NH_2_- modified TLS11a aptamer 3ʹ-NH_2_–AAAAAAA CAGCATCCCCATGTGAACAATCGCATTGTGATTGTTACGGTTTCCGCCTCATGGACGTGCTG-5ʹ were synthesized by Shanghai Sangon Biotech Co., Ltd. (Shanghai, China). Carboxy graphene quantum dots(Cat.No: XF090) were purchased from Nanjing Xianfeng nano Biotechnology Co., Ltd; Adriamycin(DOX) (Cat.No: 3010) was purchased from Shanghai Biochempartner Co., Ltd. (Shanghai, RPC).

### 2.2 Cell culture

The hepatoma cell line H22 from BALB/c mice was purchased from the China Center for Type Culture Collection (CCTCC). The cells were cultured in a 37°C with 5% CO_2_ in Dulbecco’s modified Eagles’s medium supplemented with 10% fetal bovine serum, 100 U/ml penicillin, 100 μg/ml streptomycin.

### 2.3 Animals

Female H-2Kd BALB/c nude mice (6–8 weeks, weight: 20 ± 2 g) were provided by the Animal Experimental Center of Zhejiang Chinese Medical University. The experiment was authorized by the Experimental Animal Ethics Committee of Hangzhou Medical College.

### 2.4 Preparation of DOX-Fe_3_O_4_@CS nanoparticles and assessment of drug encapsulation efficiency

In 40 mL of secondary distilled water, 2.8 mg FeSO_4_ · 7H_2_O was dissolved and 10 mL of 40 g/L PEG-2000 solution was added. The solution was stirred in a constant temperature water bath at 30°C, and 30 mL dilute ammonia water was added dropwise to form dark green Fe(OH)_2_ by adjusting the pH to 10. Next, 300 μL of H_2_O_2_ was added to oxidize part of Fe^2+^ to Fe^3+^, stirred for 10 min, and then was placed in 180°C reactors for 10 h. The reaction solution was removed and washed two times with distilled water and three times with ethanol. Magnetic Fe_3_O_4_ nanoparticles were isolated and dried at 37°C by magnetic separation. To prepare Fe_3_O_4_@CS, 0.2 g of chitosan was dissolved in 20 mL of 2% acetic acid solution. By adding K_2_CO_3_ solution at 60°C, the solution pH can be adjusted to 9. The process of producing Fe_3_O_4_ was applied to a chitosan acetic acid solution. After four hours of ultrasonic treatment at 60°C, the deposits were separated by magnets and washed numerous times with ethanol and deionized water to obtain Fe_3_O_4_@CS. Slowly, 1 mL of DOX-HCI (2 g/L) solution was added to the dispersion. The mixture was stirred at room temperature overnight. The mixture was centrifuged at high speed and washed with PBS to remove the free DOX, and the residue was dried overnight in a vacuum drying oven to obtain DOX-Fe_3_O_4_@CS.

The standard curve of DOX based on the relationship between DOX concentration in solution and UV absorbance was plotted to measure drug encapsulation efficiency (EE) and loading efficiency (DLE). The absorbance values of DOX encapsulated in Fe_3_O_4_@CS nanoparticles were measured using Uv-vis. DOX concentration was derived from the standard curve; the drug encapsulation and loading efficiency could be determined based on calculation. EE is defined as the actual doxorubicin loading of nanoparticles referred to as the inputted doxorubicin. DLE is defined as the ratio of the mass of the drug encapsulated in nanoparticles to the total mass of drug-loaded nanoparticles.

EE (%) = mass of DOX loaded/mass of DOX inputted×100%

DLE (%) = mass of DOX loaded/mass of nanoparticles ×100%

### 2.5 Preparation of DOX-Fe_3_O_4_@CS@GQD-Apt (DOX-Fe_3_O_4_@CGA) nanoparticles

The capping of GQDs and the grafting of aptamers were conduceted via the amide bond. First, 10 mg DOX-Fe_3_O_4_@CS nanoparticles and 2 mg carboxyl GQDs were added in a 2 mL MES (4-morpholineethanesulfonic acid) buffer solution (pH = 6). After shaking for three hours, the particles were crosslinked overnight using EDC (1-ethyl-3-[3-dimethylaminopropyl] carbodiimide hydrochloride)/NHS (N-hydroxysuccinimide). Then, they were centrifuged and rinsed five times with dispersion liquid. Second, 10 mL aptamer (5 mM) was added to the above solution, and the reaction was allowed to run for 2 h. Following that, 1% BSA was added to seal for 30 min, the mixture was centrifuged at high speed and washed three times, and the aptamer coupled nanoprobes (DOX-Fe_3_O_4_@CGA) were obtained via magnetic adsorption.

### 2.6 Characterization of nanoparticles

The morphology and structure of the prepared nanoparticles were examined by transmission electron microscopy (TEM, TESCAN VEGA3 LMU, Tescan USA Inc.; Cranberry Twp., PA, USA). The nanoparticles’ hydrodynamic particle size, polydispersity index (PDI) were evaluated by a Malvern Zetasizer Nano-ZS (Malvern Instruments, Worcestershire, UK). The superparamagnetic properties of the magnetite nanoparticles were performed by a vibrating sample magnetometer (VSM-7400, Lakeshore, USA). Fourier-transform infrared spectra were measured on a Nexus 670 spectrometer (Thermo Fisher Scientific). Thermogravimetric analysis (TGA) was performed with a Perkin Elmer Pyris1 thermogravimetric analyzer.

### 2.7 In vitro *DOX release*

The dialysis method was used to investigate the *in vitro* release of DOX from DOX-Fe_3_O_4_@CGA nanoparticles. Briefly, 0.2 mg of DOX-Fe_3_O_4_@CGA was dispersed in 2.0 mL of PBS and then transferred to a dialysis bag (molecular cutoff of 3000 DA). The samples were dialyzed in PBS (80 mL) at 37°C with continuous stirring at pH 5.5 or 7.4, and DOX-Fe_3_O_4_@CGA was incubated with or without NIR irradiation (808 nm, 2 W/cm,^2^ 5 min) at specified time points. At predetermined time intervals, 1 mL of release medium was removed for analysis and replaced with an equivalent volume of fresh PBS; the amount of DOX released was quantified using a UV-vis spectrophotometer; all experiments were conducted three times.

### 2.8 MRI of nanoparticles in aqueous solutions

The MRI of nanoparticles in aqueous solutions was performed using a clinical MR scanner (Skyra 3.0 T, Simens, Germany). In brief, a Fe_3_O_4_@CS@GQD solution with a Fe concentration of 0 ~ 0.8 mm was produced and aligned. The T2 * scan sequence parameters for MRI were set as follows: TR = 150 ms, TE = 40 ms, slice thickness = 10 mm. T2 values at various concentrations were obtained through signal processing with a magnetic resonance scanner and a postprocessing system. The relaxivity value (*r2*) was found by fitting the data points to a slope based on solution concentration and relaxation efficiency.

### 2.9 *Assessments of cellular uptake* in vitro

H22 logarithmic growth phase cells were harvested and cultured overnight in six-well plates at a density of 1 × 10^5^ cells per well. Following that, 2 mL of a new medium with DOX or DOX-Fe_3_O_4_@CGA (at DOX concentrations ranging from 5 to 20 μg/mL) was added to the culture medium. For 5 min, the photothermal group was exposed to a NIR laser at 808 nm at 2 W/cm.^2^ Following that, the medium was removed. The cells were washed several times with PBS before being fixed by adding 4% paraformaldehyde for 20 min, removing the paraformaldehyde, adding a suitable amount of DAPI staining for 5 min, and washing with anti fluorescence quencher. Finally, confocal fluorescence microscopy was used to examine them.

### 2.10 Cytotoxicity assay

*In vitro* cytotoxicity of Fe_3_O_4_@CGA was investigated using MTT assay. H22 cells were grown in cell incubators after being injected into 96 well plates. The medium was then removed, and 100 µL of medium containing various concentrations of Fe_3_O_4_@CGA was added. The laser group was exposed to a laser (808 nm, 2 W/cm^2^) for 5 min. After incubating for 24 h, it was rinsed three times with PBS before mixing uniformly with 100 µL of 0.5 mg/mL MTT solution. To dissolve the nail Zan crystal, 150 µL of DMSO solution was added and vibrated for 10 min. A microplate reader measured the absorbance value to calculate the relative survival rate of cells.

### 2.11 In vivo *photothermal therapeutic effect evaluation and biodistribution studies*

To establish a hepatocellular carcinoma-bearing mouse model, 2.0 × 10^6^ H22 cells were seeded into the right axilla of BALB/c mice (6–8 weeks). To examine biodistribution, DOX-Fe_3_O_4_@CGA was labeled with ICG, a commonly used fluorescent dye that can self-assemble and cross-link with CS.^30^ When tumors reached 300 mm^3^, mice were randomly assigned to one of four groups (n = 5): (1) PBS+Laser, (2) free ICG, (3) ICG labeled DOX-Fe_3_O_4_@CGA, and (4) ICG labeled DOX Fe_3_O_4_@CGA + laser. Group 1 was injected with 100 μL PBS and laser-irradiated. Group 2 received ICG injections at a dose of 2 mg/kg ICG in 200 mL of PBS per mouse. Group 3 received the same dose of DOX- Fe_3_O_4_@CGA-ICG as Group 2. Group 4 received the same DOX- Fe_3_O_4_@CGA-ICG injection as Group 3 and was laser irradiated. Following the intravenous injection, the mice were sedated with 100 ml of 1% 50 mg/kg sodium pentobarbital (intraperitoneal injection) for a standard irradiation procedure. The laser groups were irradiated for 5 min every two days with an 808 nm laser (2 W/cm^2^) under the guidance of fluorescence imaging, beginning on day 1 (after the first injection for 24 h). The Bruker imaging system (FXPro, Carestream Health, Inc, USA) was used to capture fluorescence images of groups 2, 3, and 4. During 5-min irradiation, infrared thermometers were used to record the temperature change of the tumor site in groups 1, 3, and 4.

### 2.12 In vivo *antitumor efficacy assessment*

The tumor-bearing mice were established using the method outlined above. The mice were then divided into five groups (n = 4/group): group 1 injected PBS via the tail vein, groups 2 and 3 received Fe_3_O_4_@CGA, and DOX, respectively. DOX-Fe_3_O_4_@CGA was injected into groups 4 and 5, and groups 2 and 5 were also exposed to NIR light. DOX dosage was set evenly at 8 mg/kg and administered every two days for five treatments. Tumor volume and mouse body weight were monitored every two days during therapy. The tumor volume (V) was determined using the short and long diameters (d, D) of tumor tissue (V = d^2^ × D/2). All animals were slaughtered with 180 mg/kg sodium pentobarbital after 22 days of treatment, and tumor tissues were weighed and H-E stained to assess anti-tumor activity. To evaluate the biological toxicity of treatment, major tissues from PBS and DOX-Fe_3_O_4_@CGA+Laser groups were collected for H-E staining, including heart, liver, spleen, lung, and kidneys. The tumor-bearing mice were established to measure mouse survival after treatment. Mice were divided into five groups (n = 8/group) and subjected to the previously described treatment. The survival times of mice in several groups were reported.

### 2.13 Statistical analysis

The independent samples t-test was used to determine statistically significant differences, and *p < .05 were defined as statistically significant differences. Data were presented as mean ± SD.

## 3. Results and discussion

### 3.1 Physical and chemical characteristics of nanocomposites

**Figure 1.**
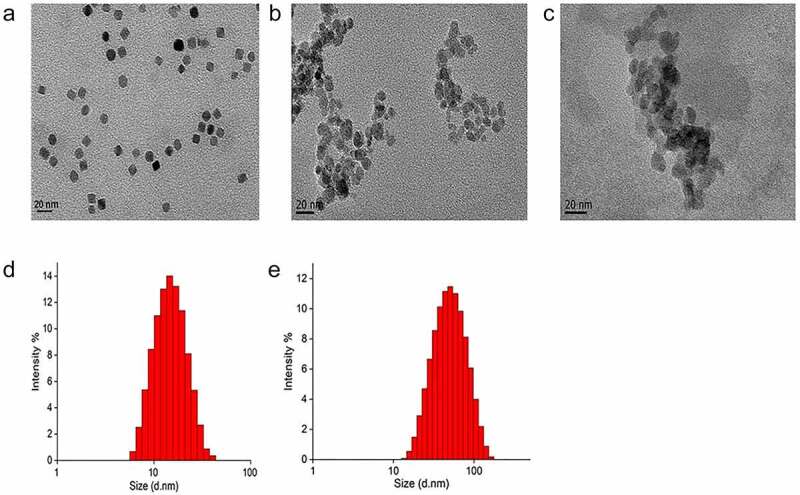
Particle size characterization of nanoparticles. TEM images of Fe_3_O_4_ (a), Fe_3_O_4_@CS (b), and DOX-Fe_3_O_4_@CGA (c). Hydrodynamic particle sizes characterization of Fe_3_O_4_@CS (d) and DOX-Fe_3_O_4_@CGA (e).

TEM was used to analyze the particle size and morphology of Fe_3_O_4_, Fe_3_O_4_@CS, and DOX-Fe_3_O_4_@CGA. As illustrated in Figure 1, the particle size of Fe_3_O_4_ was around 9 nm on average and increased to approximately 26 nm following encapsulation in CS. The average particle size increased to 37 nm after loading DOX, GQD, and aptamers. DOX-Fe_3_O_4_@CGA magnetic drug-loaded particles had a nearly spherical morphology and a uniform distribution. However, chitosan, adriamycin, aptamer, and GQD encapsulated on the surface of composite nanoparticles were not visible in TEM pictures because they were composed of components with a relatively light atomic mass, such as C, H, O, and N, and TEM was not capable of detecting such elements. The nanoparticle was further examined using DLS, IR spectroscopy, and thermogravimetry to effectively manufacture the composites. Dynamic light scattering (DLS) analysis demonstrated that Fe_3_O_4_@CS (Figure 1d) and DOX-Fe_3_O_4_@CGA (Figure 1e) were well dispersed in solution (PDI < 0.5), with wavelengths of approximately 42.2 ± 2.1 nm and 53.1 ± 2.8 nm, respectively. The hydrodynamic particle size is slightly larger than the actual size of the nanoparticles due to the influence of medium viscosity, diffusion coefficient, and so on.

**Figure 2.**
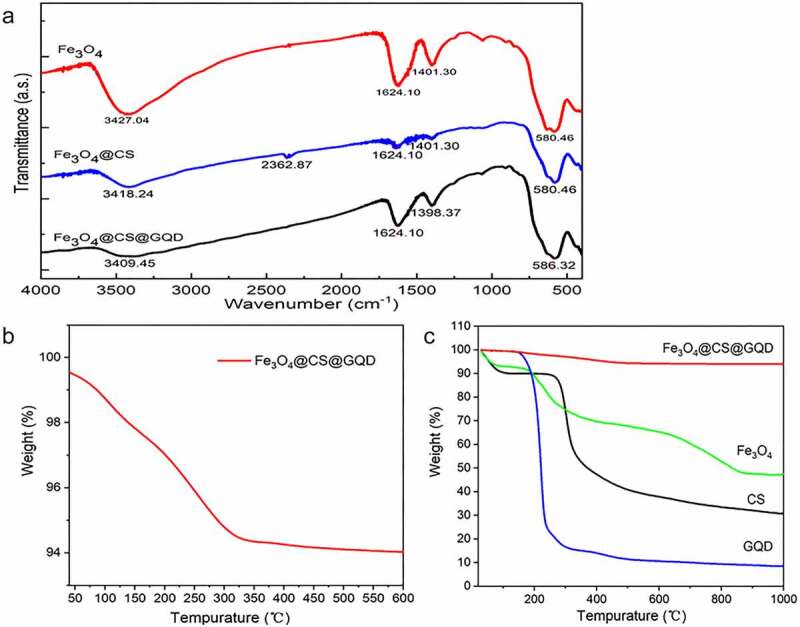
FTIR and thermogravimetric analysis of nanoparticles. (a) FTIR of Fe_3_O_4_, Fe_3_O_4_@CS, Fe_3_O_4_@CS@GQD. Thermogravimetric analysis of (b) Fe_3_O_4_@CS@GQD, and (c) Fe_3_O_4_@CS@GQD, Fe_3_O_4_, CS, and GQD.

Fourier transform infrared (FTIR) spectroscopy was used to determine the chemical functional group composition of the prepared nanomaterials. The infrared spectra (Figure 2a) revealed that the peak at 578 cm^−1^ for Fe-O group and a large peak at 3410 cm^−1^ for chitosan might be caused by O-H and NH stretching vibrations. The characteristic peak of -NH_2_ overlapped with O-H, another at 1627 cm^−1^, and another at 1646 cm^−1^ could represent the C-O stretching vibration of an incompletely deshielded acetyl group on chitosan, the C-H bending vibration at 1392 cm^−1^, and the C-O stretching rocking vibration at about 1060 cm^−1^. Additionally, Figure 2b illustrated that the first weight-loss stage occurs when Fe_3_O_4_@CS@GQD moisture is volatilized. The second stage occurs when the residual N substituent in Fe_3_O_4_@CS@GQD and C-O-C bond on the chitosan molecular chain are broken. It has been reported that graphene oxide has low thermal stability, and the carbon skeleton of graphene begins to break down abruptly at 185°C. As illustrated in Figure 2c, the residual mass percentages of the raw materials (Fe_3_O_4_, CS, and GQD) after burning are nearly identical to Fe_3_O_4_@CS@GQD, indicating that the nanocomposites were synthesized successfully.

**Figure 3.**
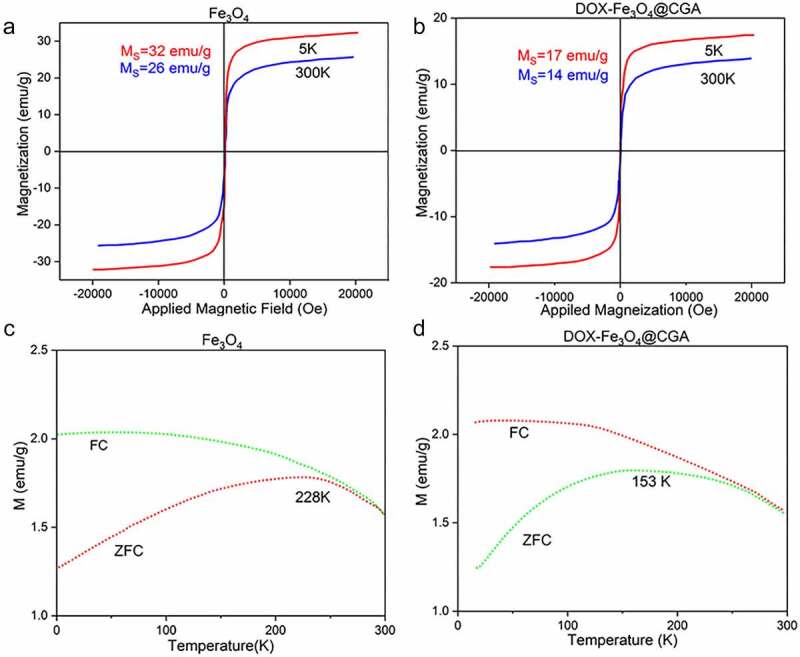
Magnetic properties testing of nanoparticles. Normalized field-dependent magnetization curves for Fe_3_O_4_ (a), DOX-Fe_3_O_4_@CGA(b) at 5 K and 300 K. Temperature-dependent magnetization curves of Fe_3_O_4_ (c), DOX-Fe_3_O_4_@CGA(d) under 50 Oe magnetic field with zero-field cooling (ZFC) and field cooling (FC).

This study aims to investigate the superparamagnetic characteristics of magnetic nanoparticles. First, the saturation magnetization intensity of Fe_3_O_4_ and DOX-Fe_3_O_4_@CGA was evaluated. As shown by the magnetization curves (Figure 3a), the magnetic intensity of Fe_3_O_4_ was 32 emu/g at 5 K and 26 emu/g at 100 K. The magnetic intensity of DOX-Fe_3_O_4_@CGA decreased to 17 and 14 emu/g due to the coating of drug-loaded chitosan and GQDs, which reduced the Fe_3_O_4_ mass percentage (Figure 3b), showing the successful synthesis of the composite drug-loaded nanomaterials produced. The variation of magnetization intensity with temperature for DOX-Fe_3_O_4_@CGA and Fe_3_O_4_ nanoparticles were investigated in a 50 Oe magnetic field with zero-field cooling (ZFC) and field cooling (FC) (FC). DOX-Fe_3_O_4_@CGA and Fe_3_O_4_ have notable peaks at 228 K and 153 K, respectively, as shown in Figure 3c, d. The nanoparticles are superparamagnetic above this temperature. At 300 K (25°C), the magnetic nano platform still has superparamagnetism, making it good for magnetic resonance imaging contrast agents.

### 3.2 In vitro *magnetic resonance imaging effects of Fe_3_O_4_@CGA*

**Figure 4.**
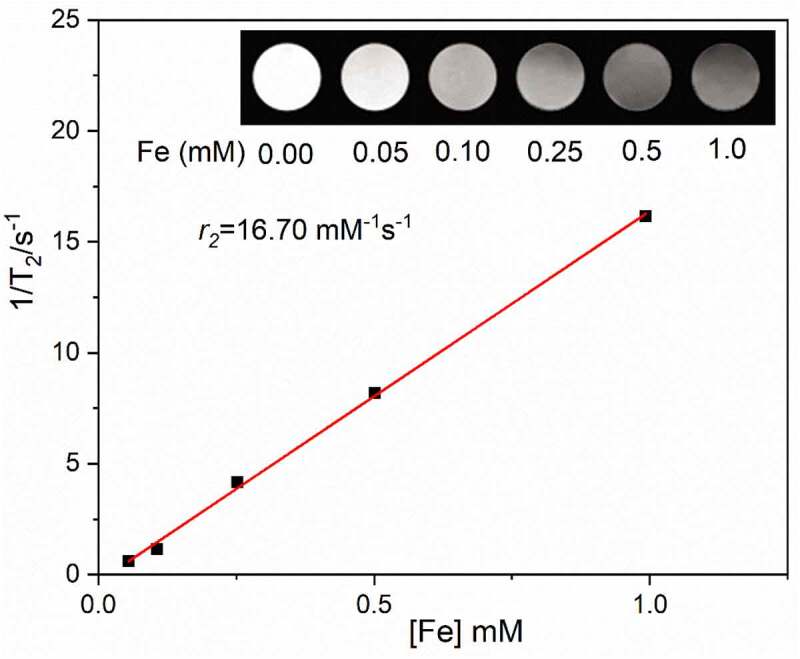
MRI of DOX- Fe_3_O_4_@CGA solution. (a) MRI images of DOX- Fe_3_O_4_@CGA solution with different Fe concentrations. (b) Plot of Fe concentration (mM) versus 1/T2 with slope indicating specific relaxation rate (r2).

A 3.0 T clinical MR scanner was used to assess the magnetic resonance imaging capacity of Fe_3_O_4_@CGA. As displayed in Figure 4, the T2 signal intensity of the nanoparticles gradually decreased with increasing Fe concentration. The specific relaxation rate (*r2*) of Fe_3_O_4_@CGA was determined to be 16.70 m/M/S after the images were processed using the MRI data processing system and fitted with a linear function.

### 3.3 DOX encapsulation efficiency and release from the nanoparticles

**Figure 5.**
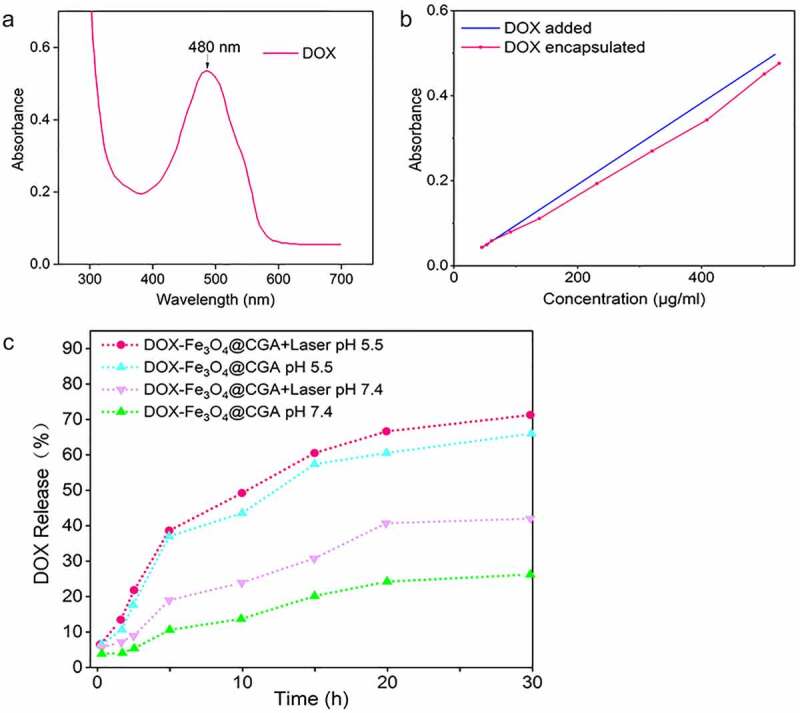
Drug encapsulation efficiency and release performance. (a) UV absorption pattern of DOX. (b) UV absorption curves of different concentrations of DOX added to the nanoparticles and DOX encapsulated. (c) Drug release profiles of DOX-Fe_3_O_4_@CGA with or without NIR laser under different pH conditions.

The encapsulation efficiency was evaluated using a UV-vis spectrophotometer. DOX’s maximum UV absorption peak, as shown in Figure 5a, was about 480 nm. Then, based on DOX concentration and UV absorbance, the standard curve of DOX was plotted. At a volume ratio of 1:10, ten solutions containing varying doses of adriamycin were added to Fe_3_O_4_@CS nanoparticles solution. As depicted in Figure 5b, the amount of DOX encapsulated has an almost linear relationship with the concentration of DOX supplied. Using the methodology described in section 2.4, the drug encapsulation efficiency was around 85%, and the loading efficiency was approximately 12% on average.

To investigate the drug release features of drug-laden nanosystems, the drug release efficiency of DOX-Fe_3_O_4_@CGA at pH 5.5 and 7.4 were evaluated with and without NIR irradiation. The drug release efficiency of drug-laden nanoparticles was lower at pH = 7.4 than at pH = 5.5, as illustrated in Figure 5c. When nanoparticles were treated with extra NIR light, the proportion of drug release in the acidic environment might reach around 70% after 30 h, compared to only approximately 20% in the neutral environment without light. It was found that both near-infrared light and slightly acidic conditions could accelerate drug release.

### 3.4 In vitro *cellular drug uptake and targeting*

**Figure 6.**
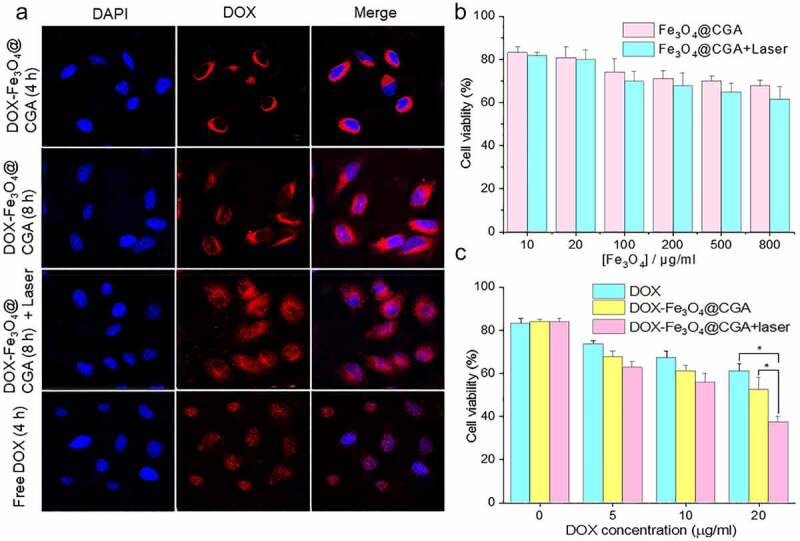
Cellular experiments of DOX-Fe_3_O_4_@CGA. (a) Fluorescence Microscope images of H22 cells treated with DOX-Fe_3_O_4_@CGA, DOX Fe_3_O_4_@CGA+Laser, and free DOX. (b) Statistical chart of cytotoxicity experiments. (c) Statistical chart of tumor cell killing experiments. *p < .05.

The cellular uptake of DOX-Fe_3_O_4_@CGA was studied in H22 cells using laser confocal microscopy. After 4-h incubation, the red fluorescence of free DOX was detected in the nucleus, as presented in Figure 6a. However, DOX was mostly found in cytoplasmic lysosomes in DOX-Fe_3_O_4_@CGA group. When the incubation time was increased to eight hours, the red fluorescence became brighter, but most still did not co-localize with the nucleus. The NIR laser-triggered drug release of DOX from DOX-Fe_3_O_4_@CGA in H22 cells was further investigated. The cells were exposed to an 808 nm laser for (5 min, 2 W/cm^2^) before being incubated for four h. In the figure, there was a strong red fluorescence in the nucleus. The results show that DOX-Fe_3_O_4_@CGA maintained its perinuclear localization throughout the experiment and that DOX migrated away from the nanoparticles and into the nucleus in response to NIR laser.

### 3.5 Cytotoxicity assay

To evaluate the biocompatibility of Fe_3_O_4_@CGA nanocarriers, the cell viability of Fe_3_O_4_@CGA on H22 cells was detected at a range of Fe ion concentrations by the MTT (3-(45)-dimethylthiahiazo (-z-y1)-35-di- phenytetrazoliumromide) technique. Figure 6b demonstrated that the toxicity of Fe_3_O_4_@CGA on H22 cells was low, and cell survival was greater than 70% even at high doses (800 µg/mL), indicating excellent biocompatibility of Fe_3_O_4_@CGA. The cell viability in Fe_3_O_4_@CGA+Laser group was decreased due to the photothermal effect of nanocarriers, although it remained greater than 65%. The cytotoxic effect of various treatments after loading DOX on nanocarriers was explored further (Figure 6c). Because of its tumor cell targeting effect, DOX-Fe_3_O_4_@CGA group was more effective in killing tumor cells than DOX group. The DOX-Fe_3_O_4_@CGA+Laser group outperformed DOX or DOX-Fe_3_O_4_@CGA groups in terms of tumor cytotoxicity (*p* < .05). When DOX-Fe_3_O_4_@CGA+Laser group was supplied with a DOX concentration of 20 µg/ml, cell viability dropped to 37%.

### 3.6 In vivo *imaging and biodistribution*

**Figure 7.**
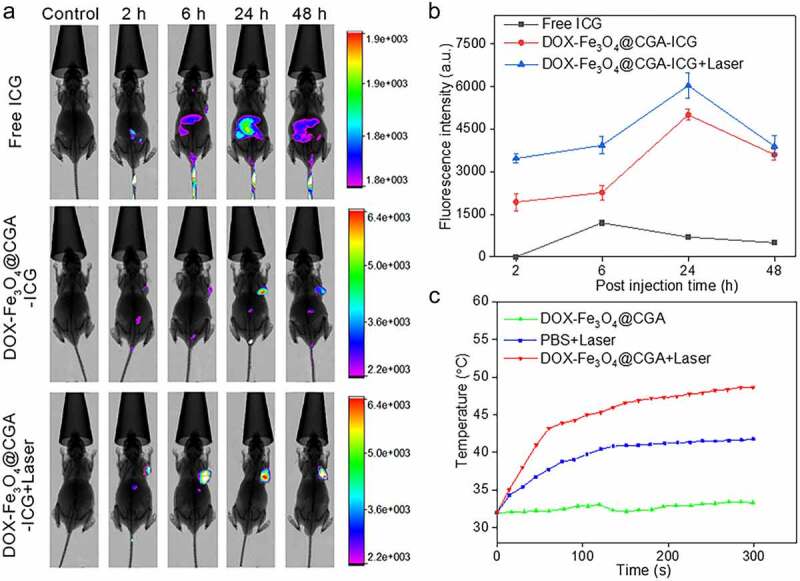
Photothermal properties of the DDS and *in vivo* distribution. (a) Examining the distribution of DOX-Fe_3_O_4_@CGA using *in vivo* imaging systems. (b) Fluorescence intensity at the tumor site of each group. (c) Temperature changes of the mouse tumor tissues during treatment.

In Figure 7a, samples of the tumor regions were collected at indicated periods (2, 6, 24, and 48 h) for the biodistribution of ICG labeled DOX-Fe_3_O_4_@CGA in the H22 tumor-bearing mice, and a statistical graph of the fluorescence intensity at the tumor site is depicted in Figure 7b. Fluorescence was detectable in the H22 tumor and liver at 6 h for ICG group, but it was virtually eliminated at 24 h. The fluorescence intensity in the liver was lower in DOX-Fe_3_O_4_@CGA-ICG group. On the other hand, the fluorescence intensity gradually increased at the tumor site. It had the highest fluorescence intensity at 24 h after injection, then decreased significantly at 48 h, most likely due to the degradation of nanomaterials by metabolism in mice. When the fluorescence intensity was coupled with infrared light, it was higher at the tumor site after injection, implying that the photothermal boosted drug-laden particles aggregated at the tumor site, treating the tumor more effectively.

### 3.7 In vivo *synergistic photothermal-chemotherapy*

To determine the susceptibility of mice to various treatments. Temperature changes at the tumor location of mice were monitored using an infrared thermometer. As illustrated in Figure 7c, DOX-Fe_3_O_4_@CGA group without NIR exhibited relatively minor temperature fluctuations during 5 min. When the tumor site was exclusively bombarded with NIR, the temperature rose from 38°C during the first 80 seconds to 41°C. Within the first 50 seconds, the temperature in the photothermal group increased rapidly to roughly 43°C, then gradually increased to 48°C near the tumor site. Even though the tumor cells are killed at this temperature, the distance between the laser probe and the mice was changed to keep the tumor site temperature between 43°C and 45°C. This allowed for gentle photothermal treatment without causing damage to normal tissues.

**Figure 8.**
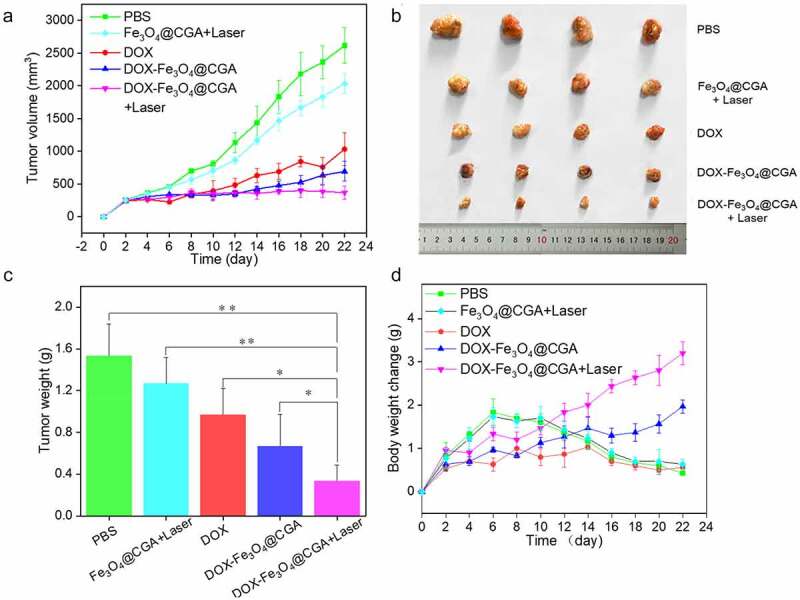
*In vivo* antitumor efficacy. (a) The tumor growth curves of different groups. (b) Photographs of the tumors harvested and (c) tumor weights after different treatments. *p < .05, **p < .01. (d) Bodyweight changes of different groups of mice.

**Figure 9.**
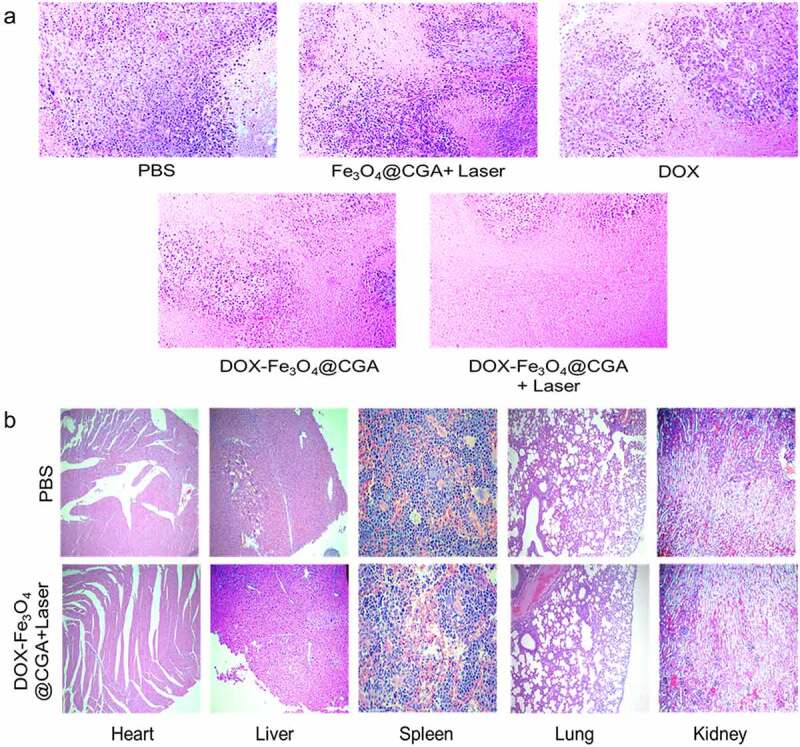
HE-stained images of tissues from (a) tumors after different treatments, and (b) major organs (heart, liver, spleen, lung, and kidneys) of PBS and DOX-Fe_3_O_4_@CGA+Laser groups.

The tumor volume, tumor weight, mouse body weight, and survival rate during therapy were evaluated to determine the anti-tumor impact of DDS *in vivo*. As illustrated in Figure 8a, the tumor volume of each group increased over time, with the PBS group exhibiting rapid tumor growth during treatment. The tumor grew faster in the Fe_3_O_4_@CGA+Laser group than in the other three, demonstrating that photothermal therapy alone was ineffective. This could be because the photothermal therapy applied in this study was moderate. The DOX group developed tumors slowly for the first eight days, similar to DOX-Fe_3_O_4_@CGA+Laser and DOX-Fe_3_O_4_@CGA groups; the rapid growth in the latter period may be attributed to drug-resistance development in the animals and ineffective treatment targeting. Due to the synergistic therapeutic impact of photothermal materials, DOX-Fe_3_O_4_@CGA+Laser group had smaller tumor volumes than DOX-Fe_3_O_4_@CGA group during treatment. From the eighth day on, DOX-Fe_3_O_4_@CGA group demonstrated an advantage over DOX group, most likely due to tumor targeting by TLS11a aptamer and EPR effect of nanoparticles. Figure 8b depicts the tumor tissue after the treatment. As shown in Figure 8c, the average tumor weight of DOX-Fe_3_O_4_@CGA+Laser group was much less than that of the other groups. Bodyweight is another useful indicator of the health status of mice and can be used to determine the efficacy of photothermal treatment. As shown in Figure 8d, during the pre-treatment period (within eight days), the mice in PBS group gained weight faster due to faster tumor growth. The mean bodyweight of DOX-Fe_3_O_4_@CGA+Laser and DOX-Fe_3_O_4_@CGA groups increased gradually, indicating a gradual return to health following tumor growth control. PBS and DOX groups were in poor condition and lost significant weight as the tumors progressed. HE staining was utilized to examine organ and tumor damage in mice. As illustrated in Figure 9a, DOX and Fe_3_O_4_@CGA+Laser groups killed only a few cancer cells. Concurrently, DOX-Fe_3_O_4_@CGA group eliminated the most cancer cells. Almost all tumor cells were killed in the DOX-Fe_3_O_4_@CGA+Laser group, and visible degenerative changes such as nuclear hemorrhage and nuclear lysis can be seen. As illustrated in Figure 9b, no significant morphological alterations were observed in the organs of PBS or experimental groups, indicating that the synergistic treatment was not harmful.

**Figure 10.**
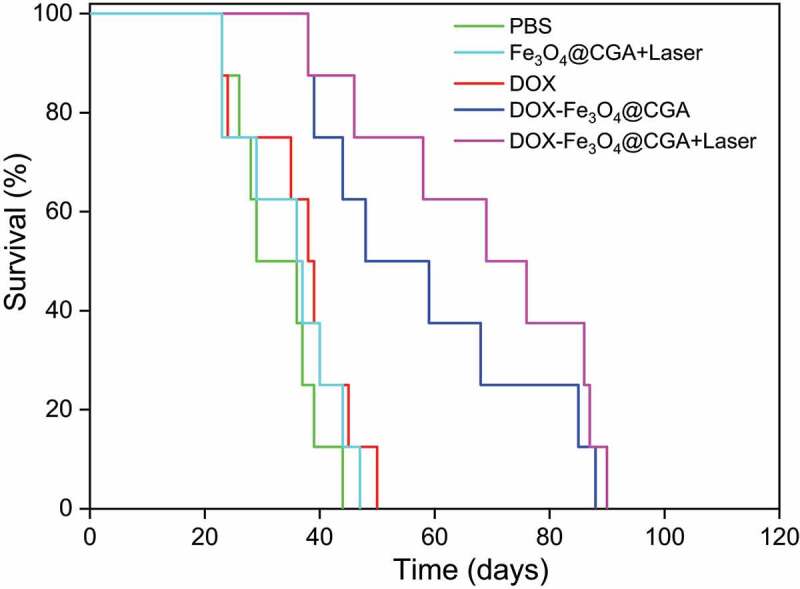
Survival curve of different groups of mice.

Additionally, the survival time of mice was evaluated under various interventions. Kaplan-Meier curves (Figure 10) revealed the median survival times for the PBS, Fe_3_O_4_@CGA+Laser, DOX, DOX-Fe_3_O_4_@CGA, and DOX-Fe_3_O_4_@CGA+Laser groups were 36, 36, 39, 59, and 76 days, respectively. The median survival time in Fe_3_O_4_@CGA+Laser group was comparable to that in the control group, which could be attributed to the favorable photothermal treatment. In comparison, DOX-Fe_3_O_4_@CGA+Laser group dramatically increased the median survival time of mice, demonstrating that photothermal from DDS successfully promoted the chemotherapeutic effect. The DOX group had a shorter median survival time than DOX-Fe_3_O_4_@CGA and DOX-Fe_3_O_4_@CGA+Laser groups. It demonstrated that DDS is less toxic and effective against tumors.

## 4. Conclusions

In summary, a unique DDS based on GQDs and CS has been designed successfully. Due to EPR effect and HCC cell-specific aptamers, DDS targeted HCC both actively and passively. Due to superior photothermal capabilities of GQDs and appropriate surface modification, they may efficiently generate heat in the near-infrared to promote the aggregation of magnetic chitosan drug carriers in tumors and acid-sensitive drug release. Meanwhile, it found no evidence of substantial biological toxicity or adverse effects *in vivo* or *in vitro* by varying the size of the synthesized material and the intensity of photothermal therapy. Overall, the constructed DDS is biocompatible and multifunctional, and this study may provide a new technique for future research on photothermal chemotherapy paired with HCC.
